# The Synthetic Phenotype of Δ*bamB* Δ*bamE* Double Mutants Results from a Lethal Jamming of the Bam Complex by the Lipoprotein RcsF

**DOI:** 10.1128/mBio.00662-19

**Published:** 2019-05-21

**Authors:** Elizabeth M. Hart, Meera Gupta, Martin Wühr, Thomas J. Silhavy

**Affiliations:** a Department of Molecular Biology, Princeton University, Princeton, New Jersey, USA; b Department of Chemical and Biological Engineering, Princeton University, Princeton, New Jersey, USA; c Lewis-Sigler Institute for Integrative Genomics, Princeton University, Princeton, New Jersey, USA; University of Michigan-Ann Arbor

**Keywords:** bacterial genetics, Bam complex, *Escherichia coli*, OMP assembly, Rcs stress response, RcsF, outer membrane biogenesis

## Abstract

The selective permeability of the Gram-negative outer membrane (OM) is maintained by integral β-barrel outer membrane proteins (OMPs). The heteropentomeric β-barrel assembly machine (Bam) folds and inserts OMPs into the OM. Coordination of the essential proteins BamA and BamD is critical for OMP assembly and therefore the viability of the cell. The role of the nonessential lipoproteins BamBCE has yet to be characterized; however, genetic evidence suggests that they have nonoverlapping roles in OMP assembly. In this work, we quantify changes of the proteome in the conditional lethal Δ*bamB* Δ*bamE* double mutant. We show that cells lacking BamB and BamE have a global OMP defect that is a result of a lethal obstruction of an assembly-competent Bam complex by the lipoprotein RcsF. RcsF is a stress-sensing lipoprotein that is threaded through the lumen of abundant β-barrel OMPs by the Bam complex to expose the amino terminus on the cell surface. We demonstrate that simply removing this lipoprotein corrects the severe OMP assembly defect of the double mutant nearly as efficiently as a previously isolated suppressor mutation in *bamA*. We propose that BamB and BamE play crucial, nonoverlapping roles to coordinate the activities of BamA and BamD during OMP biogenesis.

## Abstract (precis)

Protein assembly into lipid bilayers is an essential process that ensures the viability of diverse organisms. In Gram-negative bacteria, the heteropentomeric β-barrel assembly machine (Bam) folds and inserts proteins into the outer membrane. Due to its essentiality, outer membrane protein (OMP) assembly by the Bam complex is an attractive target for antibiotic development. Here, we show that the conditional lethal phenotype of a mutant lacking two of the three nonessential lipoproteins, BamB and BamE, is caused by lethal jamming of the stripped-down Bam complex by a normally surface-exposed lipoprotein, RcsF. The heterotrimeric Bam complex (BamA, BamD, BamC) is nearly as efficient as the wild-type complex in OMP assembly if RcsF is removed. Our study highlights the importance of BamB and BamE in regulating the interaction between BamA and BamD and expands our understanding of the role of the Bam complex in outer membrane biogenesis.

## INTRODUCTION

Integral outer membrane proteins (OMPs) of Gram-negative bacteria such as Escherichia coli are assembled by the Bam complex, which includes a β-barrel protein, BamA, and four lipoproteins, BamBCDE (1, 2). Only BamA and BamD are essential for survival of the organism (3, 4), and the fundamental importance of this assembly machine is evidenced by the fact that homologues of BamA are found in both mitochondria and chloroplasts (5, 6). The role of the nonessential lipoproteins, BamBCE, in bacteria is unclear. E. coli mutants lacking any one of the nonessential lipoproteins exhibit modest to nearly undetectable defects in the permeability of the outer membrane (OM), with *bamB* mutants showing greater defects than either *bamC* or *bamE* mutants (7, 8). It seems likely that these proteins increase the efficiency of the Bam complex, allowing faster OMP assembly and thus faster growth of the organism. However, the molecular mechanism by which these proteins increase efficiency of the Bam complex has not been established.

Mutants that lack both BamB and BamE exhibit severe growth defects; these double mutants will not grow in rich media and propagate best at low temperatures (7, 9). It has been proposed that this conditional lethality is because these two lipoproteins perform redundant functions (9). Alternatively, the loss of BamB and BamE may simply decrease the efficiency of the Bam complex to a degree that inhibits growth. Another explanation is that these nonessential lipoproteins have evolved to perform specialized functions and when both these functions are compromised, the assembly of certain β-barrel proteins is more strongly affected than others. Evidence suggests that the accumulation of unfolded OMPs in the periplasm is a lethal event, and it is likely that any subset of unfolded OMPs is just as lethal as any other (10). Moreover, if the subset of affected proteins includes LptD, the essential OMP required for the assembly of lipopolysaccharide (LPS) on the cell surface, the cell may die from a severely compromised OM (11).

Several lines of evidence suggest that the nonessential Bam lipoproteins may have specific functions. Cells lacking BamB exhibit severe defects in the assembly of high-volume substrates such as the generalized porins and the maltoporin LamB but not substrates such as LptD and TolC (12–14). Cells lacking BamE fail to assemble the RcsF/OMP complexes that allow exposure of the amino-terminus of RcsF on the cell surface (15, 16). Given the extremely wide diversity of OMP substrates that are assembled by the Bam complex, it would not be surprising that certain Bam lipoproteins have special activities that are critical for certain substrates.

Several different suppressors that restore normal growth of Δ*bamB* Δ*bamE* double mutants have been reported. One of these suppressors, *rpoE-S2R*, is located in the σ^E^ stress response pathway. This mutation changes the induction kinetics of the σ^E^ pathway to allow for a faster and more robust stress response. This enhanced signaling counters the elevated periplasmic stress that occurs when the Δ*bamB* Δ*bamE* double mutant is exposed to conditions that stimulate a high growth rate (17). Another set of suppressors alter residues in BamA, including the gain-of-function mutation *bamA_F494L_* (9). These mutations increase the flexibility of extracellular loop 6 and are thought to bias the conformation of BamA toward an assembly-competent state (18, 19).

These same *bamA* suppressors also restore assembly of a defective mutant of the OM LPS insertase, *lptD_Y721D_*. This mutation alters a conserved residue near the carboxy terminus of LptD that affects its recognition by BamD (19). The dual suppression of Δ*bamB* Δ*bamE* and *lptD_Y721D_* by the *bamA* suppressor mutations suggests that the assembly defects caused by the lack of both BamB and BamE and by LptD^Y721D^ are similar.

In order to probe the assembly defect caused by the simultaneous deletion of the two nonessential lipoproteins more precisely, we have employed quantitative proteomics to investigate the protein landscape of the Δ*bamB* Δ*bamE* double mutant. Our results show that cells lacking BamB and BamE exhibit a general defect in OMP assembly that is not limited to a specific subset of substrates. However, the study also led to the surprising discovery that a significant portion of the assembly block in the double mutant is due to the inactivation of the crippled Bam complex by the lipoprotein RcsF. The impaired Bam complex lacking BamB and BamE cannot assemble the RcsF/OMP complex and remains tied up in an abortive effort to do so. Simply removing RcsF restores OMP assembly almost as effectively as the *bamA_F494L_* suppressor. These results establish nonoverlapping activities for both BamB and BamE in the assembly of the interlocked RcsF/OMP complex.

## RESULTS

### Quantitative proteomic analysis of the conditional lethal Δ*bamB* Δ*bamE* mutant.

**FIG 1:**
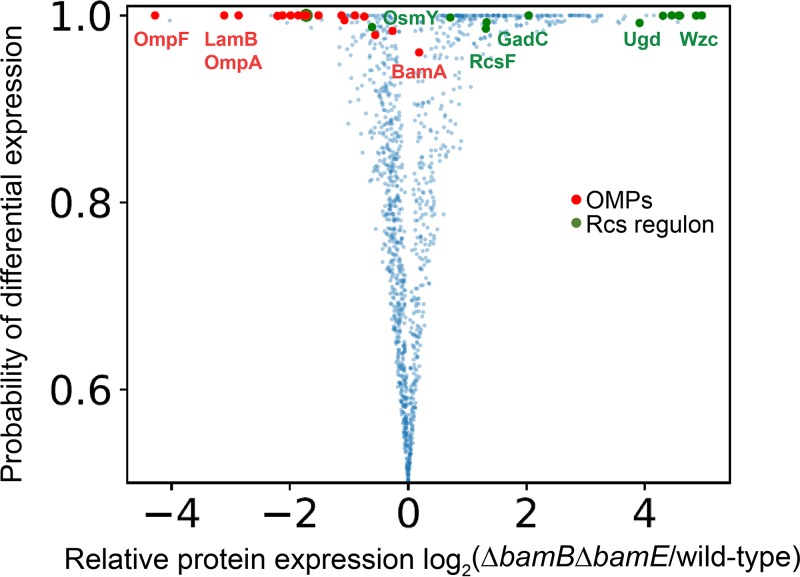
Quantitative proteomic analysis of Δ*bamB* Δ*bamE* mutant. Volcano plot of protein expression comparison between wild-type and Δ*bamB* Δ*bamE* cells. Each dot represents a protein. The horizontal axis shows the log_2_ fold change of protein abundance. The vertical axis represents the likelihood that differential expression is observed between the two conditions (21). Red dots represent β-barrel OMPs, and green dots indicate proteins involved in the Rcs stress regulon that confidently (>0.95) change their respective expression levels. Global levels of β-barrel OMPs are reduced while levels of Rcs regulon members are mostly upregulated in the Δ*bamB* Δ*bamE* mutant relative to wild type, with a high degree of confidence.

The previous isolation of a number of *bamA* mutations that suppress both Δ*bamB* Δ*bamE* and *lptD_Y721D_* led us to wonder if the two conditions arise from a common defect. To identify this impairment, we utilized quantitative multiplexed proteomics to examine the protein expression profiles of the Δ*bamB* Δ*bamE* double mutant with respect to the wild-type control. Specifically, we relied on the TMTc+ method, which utilized the complement reporter ions for accurate and precise quantification (20). Figure 1 shows a volcano plot of this proteomic data set. Statistical significance of changes was determined via the BACIQ software (21).

We have found that the levels of the majority of OMPs were significantly lowered in the double mutant compared to the wild type. This trend does not appear to correlate with OMP function or structure; rather, global OMP levels are lowered. The exception to this universal decrease is BamA, which appears to be slightly upregulated, though only with moderate confidence (Fig. 1).

We utilized GO term enrichment analysis to identify protein groups that were significantly upregulated between the double mutant and the wild-type control (22, 23) (see Table S1 in the supplemental material). Intriguingly, we identified an enrichment of proteins involved in colanic acid biosynthesis in the Δ*bamB* Δ*bamE* mutant and found that Rcs regulon members were highly upregulated in the proteomic data set of the double mutant compared to the wild type (Fig. 1). Furthermore, the Δ*bamB* Δ*bamE* mutant is mucoid when grown on minimal medium supplemented with glucose, indicating that the cells are producing and excreting capsule (24, 25). These results demonstrate that the Rcs stress response pathway is activated in cells lacking both BamB and BamE.

10.1128/mBio.00662-19.6 TABLE S1 GO term analysis of significantly upregulated proteins identified by quantitative proteomics. *, GO term biological process enrichment analysis on significantly upregulated proteins (>0.95) identified in the Δ*bamB* Δ*bamE* double mutant compared to wild type. Terms are ranked in descending order of fold enrichment. Analysis was performed using DAVID bioinformatics software (1, 2). Download Table S1, DOCX file, 0.02 MB. Copyright © 2019 Hart et al. 2019 Hart et al. This content is distributed under the terms of the Creative Commons Attribution 4.0 International license.

### Deletion of *rcsF* suppresses Δ*bamB* Δ*bamE*.

The Rcs pathway (regulator of capsule synthesis) responds to OM and peptidoglycan stress (16, 26–28). The stress sensor RcsF detects stress cues and relays the signal to the inner membrane by a mechanism that results in alleviation of repression by the negative regulator IgaA (29, 30). The inner membrane proteins RcsC and RcsD transmit the signal to the cytoplasm through a multicomponent phosphorelay system that results in phosphorylation of RcsB (25, 30–33). Homodimers of RcsB or heterodimers of RcsAB then act as transcriptional regulators to influence gene expression (34). The Rcs regulon includes genes involved in colanic acid biosynthesis, biofilm formation, and motility (25, 35, 36). While the Rcs system is a protective stress response, recent work has shown that overactivation of the Rcs system under certain conditions can be toxic (37).

**FIG 2:**
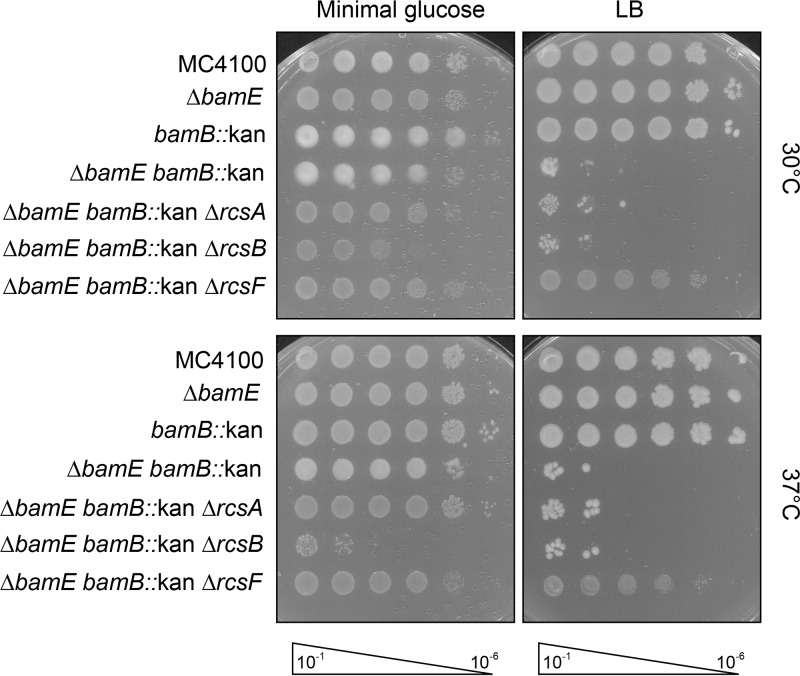
Deletion of *rcsF* suppresses Δ*bamB* Δ*bamE*. Serially diluted strains were plated on minimal glucose or rich medium at 30°C and 37°C. Deletion of *rcsF* rescues the growth defect of the Δ*bamB* Δ*bamE* mutant, while deletion of *rcsA* and *rcsB* does not.

To investigate if the Rcs pathway impedes the growth of the Δ*bamB* Δ*bamE* mutant, we deleted *rcsA*, *rcsB*, or *rcsF* and monitored growth (Fig. 2; Fig. S1). These deletions prevent expression of the Rcs regulon and stress signal input, respectively. Deletion of *rcsA*, *rcsB*, or *rcsF* had no impact on the growth of Δ*bamB* or Δ*bamE* single mutants (Fig. S1). The conditional lethal Δ*bamB* Δ*bamE* double mutant grows only on minimal medium and optimally at 30°C (7, 9). Deletion of the response regulator *rcsA* or *rcsB* does not improve the growth of cells lacking both BamB and BamE (Fig. 2). In fact, removal of RcsB impairs growth even on minimal medium, highlighting the importance of the Rcs stress response for cell viability. Remarkably, deletion of *rcsF* fully suppresses the growth phenotype of the Δ*bamB* Δ*bamE* double mutant and allows growth on rich and minimal media at high and low temperatures. These data conclusively indicate that the lipoprotein RcsF, but not the downstream Rcs signaling pathway, is responsible for the defective growth of the Δ*bamB* Δ*bamE* mutant.

10.1128/mBio.00662-19.1 FIG S1 Deletion of *rcsA, rcsB*, or *rcsF* does not cause growth defects in *bamB* or *bamE* null backgrounds. Serially diluted cultures were spotted onto minimal glucose and rich media and grown at 30°C and 37°C. (A) The *bamB*::*kan* allele in combination with the deletion of *rcsA*, *rcsB*, or both does not exhibit any growth deficiencies. (B) Deletion of *bamE*, *rcsA*, *rcsB*, or combinations of null alleles do not impact growth. Download FIG S1, TIF file, 7.1 MB. Copyright © 2019 Hart et al. 2019 Hart et al. This content is distributed under the terms of the Creative Commons Attribution 4.0 International license.

### Removal of RcsF restores global OMP levels in cells lacking BamB and BamE.

The finding that Δ*rcsF* suppresses Δ*bamB* Δ*bamE* was intriguing considering that RcsF complexes with a number of abundant OMPs, including OmpA, OmpC, and OmpF (15, 38). Site-specific cross-linking has demonstrated that the linker domain of RcsF is threaded through OMPs, resulting in localization of the lipid tail of RcsF in the outer leaflet of the OM (15). This unique conformation is folded by the Bam complex and relies on BamE for efficient assembly. When BamE is removed, RcsF stalls on BamA during assembly and levels of RcsF/OMP complexes decrease (15, 16). We posited that the simultaneous deletion of *bamB* and *bamE* exacerbates the stalling of RcsF on the Bam complex, leading to a lethal depletion of assembly-competent Bam machinery. This hypothesis would explain the global decrease in OMP levels observed in cells lacking BamB and BamE (Fig. 1). Deletion of *rcsF*, then, should remove the obstruction at the Bam complex to allow for the restoration of global OMP levels.

**FIG 3:**
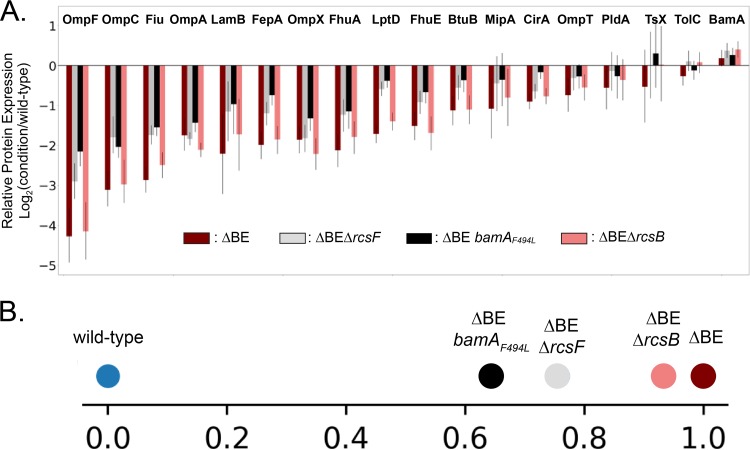
Quantitative analysis of global OMP levels. (A) The relative fold change of β-barrel OMPs comparing the Δ*bamB* Δ*bamE*, Δ*bamB* Δ*bamE* Δ*rcsF*, Δ*bamB* Δ*bamE bamA_F494L_*, and Δ*bamB* Δ*bamE* Δ*rcsB* mutants to the wild type is shown. Whiskers indicate 95% confidence intervals. The Δ*bamB* Δ*bamE* double mutant exhibits a universal defect in OMP levels with respect to the wild-type control. OMP levels are similarly reduced in the Δ*bamB* Δ*bamE* Δ*rcsB* triple mutant. Both Δ*rcsF* and *bamA_F494L_* restore OMP levels, to similar degrees. (B) Projecting the OMP protein abundance changes for various mutants on the vector defined by the differences between the wild type and Δ*bamB* Δ*bamE* double mutant indicates that Δ*bamB* Δ*bamE* Δ*rcsB* does not rescue the phenotype of the double mutant, but both Δ*bamB* Δ*bamE* Δ*rcsF* and Δ*bamB* Δ*bamE bamA_F494L_* partially rescue the OMP expression phenotype.

We utilized quantitative multiplexed proteomics (TMTc+) to compare OMP levels under the wild-type, defective (Δ*bamB* Δ*bamE,* Δ*bamB* Δ*bamE* Δ*rcsB*), and rescued (Δ*bamB* Δ*bamE* Δ*rcsF*, Δ*bamB* Δ*bamE bamA_F494L_*) conditions (Fig. 3) (20).

The Δ*bamB* Δ*bamE* mutant exhibits a universal defect in OMP levels with respect to the wild-type control. OMP levels are equivalently reduced in the Δ*bamB* Δ*bamE* Δ*rcsB* triple mutant, supporting our conclusion that the defects of the double mutant are not caused by Rcs signaling. Removal of the lipoprotein RcsF restores OMP assembly in the Δ*bamB* Δ*bamE* mutant, with the analysis showing an increase in the levels of all detected OMPs rather than only a specific set of substrates. The OMP expression levels in the Δ*bamB* Δ*bamE* Δ*rcsF* suppressed strain are very similar to the previously isolated *bamA_F494L_* suppressor. To determine the strength of suppression by Δ*rcsF*, we compared levels of all detected β-barrel OMPs. To quantify the mutants’ similarities on a global scale, we asked how the different mutants, each defined in an 18-dimensional space corresponding to the expression levels in 18 integral β-barrel OMPs, would be projected on the 1-dimensional vector that joins the defective Δ*bamB* Δ*bamE* double mutant and wild-type conditions. Consistent with the observations on the individual protein level, the two suppressed strains (Δ*bamB* Δ*bamE* Δ*rcsF* and Δ*bamB* Δ*bamE bamA_F494L_*) are very close together and the defective Δ*bamB* Δ*bamE* Δ*rcsB* is similar to the double mutant and farthest away from the wild-type control (Fig. 3B). Qualitative restoration of OMP assembly in the Δ*bamB* Δ*bamE* mutant by Δ*rcsF* was confirmed by immunoblot analysis (Fig. S2). This indicates that the suppression by Δ*rcsF* is nearly equivalent to the OMP restoration conferred by the potent *bamA_F494L_* suppressor. Together, our results illustrate that the crippled Bam complex lacking BamB and BamE can robustly assemble OMPs when RcsF is removed.

10.1128/mBio.00662-19.2 FIG S2 Deletion of *rcsF* restores OMP levels of Δ*bamB* Δ*bamE* mutant. Immunoblot analysis monitoring OMP levels in the indicated strains. All samples were grown to mid-log phase at 30°C in minimal glucose medium. Deletion of *rcsF* increases levels of OMPs in the Δ*bamB* Δ*bamE* double mutant. Download FIG S2, TIF file, 4.8 MB. Copyright © 2019 Hart et al. 2019 Hart et al. This content is distributed under the terms of the Creative Commons Attribution 4.0 International license.

### σ^E^ activation is lowered by Δ*bamB* Δ*bamE* suppressors.

**FIG 4:**
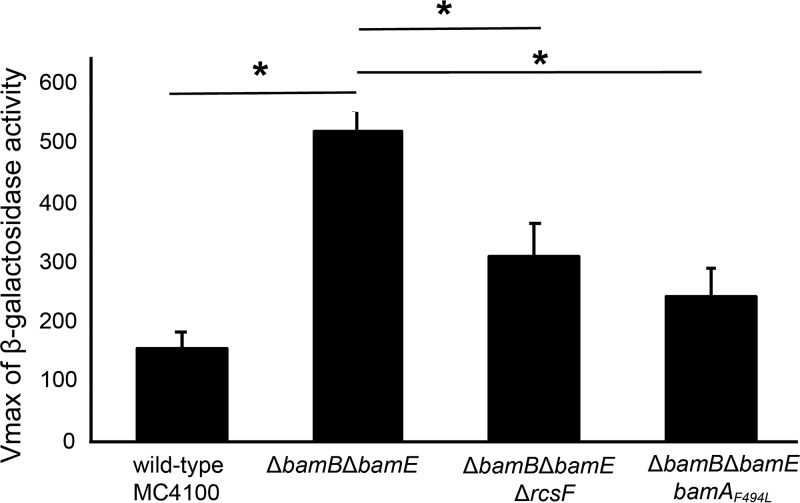
Suppressors lower σ^E^ activation of the Δ*bamB* Δ*bamE* double mutant background. σ^E^ signaling was assayed by measurement of β-galactosidase activity driven from the *rpoH*P3 promoter. All strains were grown in minimal glucose at 30°C, the permissive condition of the Δ*bamB* Δ*bamE* double mutant. *, *P* value < 0.05 as determined by *t* test. The *bamA_F494L_* and Δ*rcsF* suppressors lower σ^E^ activation of the Δ*bamB* Δ*bamE* double mutant.

Our data indicate that the Δ*bamB* Δ*bamE* double mutant suffers from an obstruction of functional Bam complexes by the lipoprotein RcsF. The σ^E^ pathway monitors OMP assembly and is activated by unfolded OMPs (39). σ^E^ activation is common under assembly-defective conditions, and a reduction in signaling serves as a measure of suppression. To further characterize the suppression of Δ*bamB* Δ*bamE* by deletion of *rcsF*, we measured σ^E^ stress response activation by β-galactosidase activity driven from a σ^E^-dependent promoter (40) (Fig. 4). The strains were tested after growth at 30°C in minimal medium supplemented with glucose, which is the permissive growth condition of the Δ*bamB* Δ*bamE* mutant.

In agreement with our hypothesis that the arrested Bam complex causes a backup of unfolded OMPs, we found that σ^E^ activity is increased in the Δ*bamB* Δ*bamE* mutant. This elevated σ^E^ signaling is reduced by both *bamA_F494L_* and Δ*rcsF* suppressor mutations. Furthermore, the lowered σ^E^ activation correlates with the strength of OMP assembly restoration as determined by quantitative proteomics (Fig. 3). Together, these data demonstrate that unfolded OMPs accumulate in the absence of BamB and BamE and that this stress is alleviated by removal of a single substrate, RcsF.

### RcsF obstruction is due to lack of BamA/D coordination during assembly.

During assembly of RcsF/OMP complexes, BamD recognizes the OMP substrate (19, 41), and BamA interacts with RcsF (15, 16, 38). Through an unknown mechanism, RcsF is then threaded through the lumen of the β-barrel OMP to anchor the amino-terminal lipidated tail in the outer leaflet of the OM (15). We hypothesized that the obstruction caused by RcsF in the absence of BamB and BamE could be due to defective interactions with either OMP-binding partners or Bam machinery.

To test if the defect arises from abnormal RcsF/OMP interactions, we removed abundant OMPs (OmpA, OmpF, or OmpC) that complex with RcsF and assayed growth (Fig. S3 and S4). Deletion of these abundant OMPs had no impact on the growth phenotypes of individual *bamB* or *bamE* mutants (Fig. S3A). Deletion of individual OMPs does not rescue the growth deficiency of Δ*bamB* Δ*bamE* cells (Fig. S3A). Furthermore, deletion of two abundant OMPs (OmpA/C, OmpA/F, or OmpC/F) had no impact on the growth of *bamB* and *bamE* single mutants and also did not suppress the growth phenotype of the double mutant (Fig. S3B and Fig. S4B). Further attempts to delete all three of the abundant OMPs in cells lacking BamE and BamB failed due to a lack of cell viability. The lack of suppression conferred by deletion of RcsF-binding partners suggests that the defect in the Δ*bamB* Δ*bamE* double mutant is specifically due to anomalous interactions of RcsF with the Bam complex.

10.1128/mBio.00662-19.3 FIG S3 Deletion of *ompA*, *ompC*, or *ompF* does not alter Δ*bamB* or Δ*bamE* growth phenotypes. Serial dilutions of overnight cultures were spotted onto minimal glucose and rich media at 30°C and 37°C to assay growth. Cells were plated on media containing 0.5% glucose when more than one OMP was deleted. Individual (A) or combinatorial (B) deletion of *ompA*, *ompC*, *ompF*, *bamE*, or *bamB* did not impede growth. Download FIG S3, TIF file, 20.2 MB. Copyright © 2019 Hart et al. 2019 Hart et al. This content is distributed under the terms of the Creative Commons Attribution 4.0 International license.

10.1128/mBio.00662-19.4 FIG S4 Deletion of OMP-binding partners of RcsF does not suppress Δ*bamB* Δ*bamE*. Efficiency-of-plating experiments were performed by serial dilution of saturated cultures onto minimal glucose or rich medium at 30°C and 37°C. Strains lacking two porins were plated on medium containing 0.5% glucose. Deletion of one (A) or two (B) OMPs that complex with RcsF, OmpA, OmpC, and OmpF does not rescue the growth defects of the Δ*bamB* Δ*bamE* mutant. Download FIG S4, TIF file, 14.7 MB. Copyright © 2019 Hart et al. 2019 Hart et al. This content is distributed under the terms of the Creative Commons Attribution 4.0 International license.

We reasoned that increasing levels of BamA may titrate RcsF away from assembly-competent complexes to allow for OMP assembly to proceed. To overexpress BamA, we utilized medium-copy-number (10 to 15 copies/cell) and low-copy-number (3 to 5 copies/cell) vectors containing *bamA* and assayed growth in the Δ*bamB* Δ*bamE* mutant background (Fig. S5A and B). Expression of BamA from either of these constructs did not improve the growth deficiency of the double mutant. Additionally, we overexpressed BamA from chromosomal loci (Fig. S5C and D). To that end, we examined suppression in *bamA* diploid cells, with BamA expressed from the native locus and at the λ attachment site, and *bamA* triploid cells, which carried an additional copy of *bamA* at the Tn*7* attachment site (Table S3). Diploid and triploid *bamA* strains did not suppress the growth defect of the Δ*bamB* Δ*bamE* mutant. We conclude that increasing levels of BamA does not suppress the defects of the conditional lethal Bam mutant. RcsF jamming of the Bam complex, then, cannot be remedied by increasing the pool of available, assembly-competent BamA.

10.1128/mBio.00662-19.5 FIG S5 BamA overexpression does not suppress Δ*bamB* Δ*bamE*. Overexpression of BamA from plasmid constructs at 10 to 15 copies/cell (A) and 3 to 5 copies/cell (B) or from chromosomal loci expressing *bamA* in diploid (C) or triploid (D) does not suppress the growth defects associated with the simultaneous deletion of *bamB* and *bamE*. Download FIG S5, TIF file, 25.6 MB. Copyright © 2019 Hart et al. 2019 Hart et al. This content is distributed under the terms of the Creative Commons Attribution 4.0 International license.

10.1128/mBio.00662-19.8 TABLE S3 Strains, plasmids, and oligonucleotides. Download Table S3, DOCX file, 0.03 MB. Copyright © 2019 Hart et al. 2019 Hart et al. This content is distributed under the terms of the Creative Commons Attribution 4.0 International license.

## DISCUSSION

Here, we show that the simultaneous deletion of *bamB* and *bamE* causes a growth defect due to a severe impairment of OMP assembly. The depleted OMP levels of the double mutant do not represent a specific subset of OMPs with a common structure or function; rather, the assembly defect is generalized and affects Bam substrates globally. The universal reduction in OMPs creates a conditional lethality that allows growth of the Δ*bamB* Δ*bamE* mutant only on minimal medium. We determine that this growth defect can be suppressed by removing a single lipoprotein, RcsF. This suppression is specific to RcsF and is not due to signaling of the Rcs stress response. Proteomic analysis demonstrates that removal of RcsF restores global OMP assembly by the impaired Bam machine and lowers the elevated σ^E^ activation of the Δ*bamB* Δ*bamE* double mutant. Strikingly, our study shows that growth deficiency of the double mutant is caused by a lethal blockage of the Bam machinery not by a β-barrel OMP, but rather by a lipoprotein. Removal of the lipoprotein RcsF allows the stripped-down Bam complex to assemble all OMPs with a surprisingly robust efficiency. Indeed, the suppressed strain grows as well as the wild type, at least under standard laboratory conditions. In the accompanying paper (42), Tata and Konovalova show that Δ*rcsF* is a powerful suppressor of all *bamE* synthetic phenotypes.

RcsF is assembled into abundant OMPs, such as OmpA, OmpC, and OmpF, by the Bam complex (15, 38). In these complexes, RcsF assumes a transmembrane topology with the lipidated amino terminus on the surface, an unstructured transmembrane domain that is threaded through the lumen of the OMP, and a periplasmic domain of known structure (15). During the assembly of this interlocked structure, RcsF initially binds to BamA while the OMP binding partner is recognized by BamD (15, 19, 38, 41). BamE is critical for the subsequent folding of the OMP around RcsF as deletion of *bamE* impedes the formation of RcsF/OMP complexes and causes an increase in the amount of RcsF bound to BamA (16). This stalling of RcsF on BamA is likely reversible because the lack of BamE causes only minor defects in overall OMP assembly (7). In the absence of BamB and BamE, however, the arrest of RcsF on BamA becomes effectively irreversible under conditions that normally support rapid growth. Our work suggests that the conditional lethality of the Δ*bamB* Δ*bamE* mutant is not due to defective interactions between RcsF and BamA or OMP-binding partners, as overexpression of BamA or removal of OmpA/C/F does not suppress the double mutant.

OMP assembly by the Bam complex relies on two essential proteins, BamA and BamD, which comprise the core of the Bam machinery (3, 4). The interaction between unfolded OMP substrates and BamD (19, 41) is communicated to BamA to promote substrate folding. Specifically, substrate engagement causes conformational changes in both BamA and BamD from a resting state to a more active state that is primed for OMP assembly (18, 19, 43, 44). Communication and conformational cycling between BamA and BamD are indispensable for the assembly of substrate OMPs, and the proteins must adopt complementary conformational states in order to function. Indeed, mutations that disrupt BamA/D coordination result in cell death (43, 44). This essential synchronization can be overcome by gain-of-function mutations in either BamA or BamD that bias the protein toward a more active conformation (19, 43). These mutations bypass the requirement for BamA/D coordination to permit the assembly of defective substrates and suppress mutations that abolish BamA/D communication (19, 43, 44). One such gain-of-function mutation is *bamA_F494L_* (19).

Previous studies have demonstrated that *bamA_F494L_* allows for the assembly of the defective substrate LptD^Y721D^ (19). The β-barrel OMP LptD also exists in a complex with a luminal lipoprotein plug, LptE (45). LptD^Y721D^ stalls on BamD during assembly and interferes with the ability of BamD to communicate with BamA. BamA^F494L^ suppresses *lptD_Y721D_* by bypassing the requirement for BamD coordination to assemble LptD^Y721D^/LptE complexes (19). Our study shows that *bamA_F494L_* suppresses Δ*bamB* Δ*bamE* to restore global OMP assembly and reduce σ^E^ activation of the double mutant. Therefore, it follows that the defect in the Δ*bamB* Δ*bamE* double mutant is also due to a disruption of BamA/D communication. In contrast to LptD^Y721D^, which is defective due to a mutant substrate bound to BamD, removal of BamB and BamE stalls the wild-type RcsF lipoprotein on BamA and prevents communication with its OMP partner bound to BamD. The failure of BamA and BamD to achieve the complementary conformations required for RcsF/OMP complex formation fatally arrests β-barrel assembly.

Our study indicates that BamB and BamE have nonoverlapping roles in RcsF/OMP assembly. BamE plays a unique role in the assembly of this interlocked complex that involves coordination of BamD bound to an abundant OMP with RcsF-bound BamA. Removal of the RcsF substrate prevents this irreversible arrest to allow for OMP assembly to proceed. As shown in the companion paper, the gain-of-function suppressor mutation *bamA_F494L_* does not restore RcsF/OMP assembly; rather, it prevents BamA/RcsF and BamD from to engaging improperly. Thus, the *bamA_F494L_* Δ*bamB* Δ*bamE* mutant phenocopies the *bamA*^+^ Δ*bamE* mutant (42)*;* RcsF/OMP complexes are not assembled under any condition when BamE is lacking. In contrast to RcsF, the lipidated amino terminus of LptE remains in the inner leaflet of the OM. Furthermore, BamA^F494L^ is able to assemble LptD^Y721D^/LptE complexes despite the absence of BamE, as evidenced by the viability of *bamA_F494L_ lptD_Y721D_* Δ*bamE* cells (see Table S2 in the supplemental material). Based on this distinction, we suggest that BamE is specifically required for the translocation of the lipidated amino terminus of RcsF to the cell surface. Unlike BamE, BamB does not play a critical role in RcsF/OMP complex assembly (16). We propose that BamB monitors the quality of OMP substrates presented by BamD to BamA prior to Bam complex engagement. Indeed, a number of assembly-defective substrates have synthetically lethal genetic interactions with *bamB* null mutations, and *bamB* mutants are defective in the assembly of high-volume OMP substrates (14, 46). In the absence of BamE, BamB monitors the RcsF/OMP complex assembly process and prevents lethal stalling of the substrate. How these two nonessential lipoproteins perform these specialized functions is a fascinating subject for further investigation.

10.1128/mBio.00662-19.7 TABLE S2 *lptD_Y721D_* Δ*bamE* synthetic lethality is suppressed by *bamA_F494L_*. *, P1 vir lysates carrying *bamE*::*kan nadB*::Tn*10*-linked alleles were transduced into the indicated strains. Transductions were plated onto medium containing tetracycline to select for successful transduction of *nadB*::Tn*10* and ampicillin to maintain the plasmid containing the *lptD* alleles. Tet^r^ transductants were then tested for Kan^r^ to calculate cotransduction frequency of both *bamE*::*kan* and *nadB*::Tn*10* alleles. The cotransduction frequency represents three separate transductions. Download Table S2, DOCX file, 0.01 MB. Copyright © 2019 Hart et al. 2019 Hart et al. This content is distributed under the terms of the Creative Commons Attribution 4.0 International license.

## MATERIALS AND METHODS

### Bacterial strains.

All strains used in this study are presented in Table S3 in the supplemental material. Strains were constructed using standard microbiological techniques and grown as previously described (47). Unless otherwise noted, all strains were grown in M63 medium supplemented with glucose at 30°C. M63 minimal glucose medium was made by supplementing M63 medium with 0.2% glucose, 1 mM MgSO_4_, 100 μg/ml thiamine, and 0.05% LB. Deletion alleles originated from the Keio collection (48). Clean deletions were made using the FLP recombinase method, as previously described (49).

### Quantitative proteomics sample preparation.

Samples were prepared as previously described with minor modifications (50). E. coli cells were grown in suspension to exponential phase (OD_600_, ∼0.5 to 0.8) in M63 minimal glucose medium at 30°C. Samples were normalized by OD_600_. Cells were harvested by pelleting at room temperature and flash frozen. Each pellet containing ∼140 μg (∼9 × 10^8^ cells) of total protein was resuspended in 100 μl of lysis buffer containing 50 mM HEPES, pH 7.2, 2% CTAB (hexadecyltrimethylammonium bromide), 6 M GuaCl (guanidine hydrochloride), and 5 mM DTT. Cells were lysed by sonication of 10 pulses, 30 s, at 60% amplitude and by further heating the lysate at 60°C for 20 min.

Next, 200 μl of lysate from every condition was methanol-chloroform precipitated (51). Protein concentration was determined using the bicinchoninic acid (BCA) protein assay (Thermo Fisher). The samples were resuspended in 6 M guanidine chloride in 10 mM 4-(2-hydroxyethyl)-1-piperazinepropanesulfonic acid (EPPS), pH 8.5, with a subsequent dilution to 2 M guanidine chloride in 10 mM EPPS, pH 8.5, for digestion with Lys-C (Wako, Japan) at room temperature with 20 ng/μl Lys-C overnight. The samples were further diluted to 0.5 mM guanidine chloride in 10 mM EPPS, pH 8.5, and digested with 20 ng/μl Lys-C and 10 ng/μl trypsin at 37°C overnight.

The digested samples were dried using a vacuum evaporator at room temperature and taken up in 200 mM EPPS, pH 8.0, for a pH shift which is necessary for optimal labeling conditions. Ten microliters of total material from each condition was labeled with 3 μl of 20 ng/μl TMT. TMT reagents were dissolved in anhydrous acetonitrile. TMT samples were labeled for 2 h at room temperature. Labeled samples were quenched with 0.5% hydroxylamine solution (Sigma, St. Louis, MO). Samples from all conditions were combined into one tube, acidified with 5% phosphoric acid (pH < 2), and subjected to a subsequent spin at 16,000 relative centrifugal force (RCF) for 10 min at 4°C. The samples were dried using a vacuum evaporator at room temperature. Dry samples were taken up in high-pressure liquid chromatography (HPLC)-grade water and stage tipped for desalting (52). The samples were resuspended in 1% formic acid to 1 μg/μl, and 1 μg of the total combined sample was analyzed with the TMTc+ approach (20).

### LC-MS analysis.

Approximately 1 μl per sample was analyzed by LC-MS. LC-MS experiments were performed on an Orbitrap Fusion Lumos (Thermo Fisher Scientific). The instrument was equipped with an Easy-nLC 1200 HPLC pump (Thermo Fisher Scientific). For each run, peptides were separated on a 100-μm-inner-diameter microcapillary column, packed first with approximately 0.5 cm of 5-μm BEH C_18_ packing material (Waters) followed by 30 cm of 1.7-μm BEH C_18_ (Waters) followed by 30 cm of 1.7-μm ethylene bridged hybrid (BEH) C_18_ (Waters). Separation was achieved by applying a 4.8% to 24% acetonitrile gradient in 0.125% formic acid and 2% DMSO over 120 min at 350 nl/min at 60°C. Electrospray ionization was enabled by applying a voltage of 2.6 kV through a microtee at the inlet of the microcapillary column. We used the Orbitrap Fusion Lumos with a TMTc+ method (20). The instrument was operated in data-dependent mode with a scan range of 500 to 1,400 *m/z* with an RF lens (%) of 60, automatic gain control (AGC) target of 1.0e6, and a maximum injection time of 100 ms. Only charge states of 2+ were included. A dynamic exclusion window of 60 s with a mass tolerance of ±10 ppm was used. Peptides with a minimum intensity of 3e6 or higher were subjected to an MS2 scan using an isolation window of 0.4 Th (or of different size if indicated) using the quadrupole. Peptides were fragmented using higher energy collisional dissociation (HCD) energy of 32%, and a mass spectrum was acquired using the Orbitrap with a resolution of 60,000 with an AGC target of 5.0e5 and a maximum injection time of 120 ms. The scan range of the Orbitrap was Auto: *m/z* High.

### MS data analysis.

A suite of software tools developed in-house was used to convert mass spectrometric data from the Thermo RAW file to the mzXML format, as well as to correct erroneous assignments of peptide ion charge state and monoisotopic *m/z* (53, 54). Assignment of MS2 spectra was performed using the SEQUEST algorithm by searching the data against the appropriate proteome reference data set acquired from UniProt, including common contaminants like human keratins and trypsin (53, 55). This forward database component was followed by a decoy component which included all listed protein sequences in reversed order. Searches were performed using a 20-ppm precursor ion tolerance, where both peptide termini were required to be consistent with trypsin or LysC specificity, while allowing one missed cleavage. Fragment ion tolerance in the MS2 spectrum was set at 0.02 Th (TMTc+), TMT was set as a static modification of lysine residues and peptides’ N termini (+229.162932 Da), and oxidation of methionine residues (+15.99492 Da) was set as a variable modification. An MS2 spectral assignment false discovery rate of 0.5% was achieved by applying the target decoy database search strategy (54). Filtering was performed using a linear discrimination analysis with the following features: SEQUEST parameters XCorr and unique ΔXCorr, absolute peptide ion mass accuracy, peptide length, and charge state. Forward peptides within 3 standard deviation of the theoretical *m/z* of the precursor were used as a positive training set. All reverse peptides were used as a negative training set. Linear discrimination scores were used to sort peptides with at least seven residues and to filter with the desired cutoff. Furthermore, we performed a filtering step toward the protein level by the “picked” protein false-discovery rate (FDR) approach (56). Protein redundancy was removed by assigning peptides to the minimal number of proteins which can explain all observed peptides, with above-described filtering criteria (57, 58). We did not use isolation specificity filtering for the TMTc+ method, as coisolation of other peptides does not perturb the measurement results for this method (20). The probabilities of differential expression were calculated based on agreement between the underlying peptides and signal level of the peptides for every protein quantified in the experiment using the BACIQ software (21).

GO term enrichment analysis of significantly upregulated proteins from the quantitative proteomics data set (significance cutoff score = 0.95) was performed using the DAVID bioinformatics software (22, 23). Biological process GO term categories were ranked by fold enrichment, and the *P* value was listed as determined by DAVID. Classification of proteins as β-barrel OMPs or members of the Rcs stress regulon was determined using the EcoCyc database (59).

### Efficiency-of-plating (EOP) assay.

Overnight cultures were grown in M63 minimal glucose medium at 30°C. Cultures were normalized by absorbance at 600 nm. Tenfold serial dilutions were made in M63 minimal glucose, plated onto M63 minimal glucose or LB agar, and incubated at 30°C or 37°C. Plates were supplemented with chloramphenicol (20 μg/ml), arabinose (0.2%), or fucose (0.05%) where indicated.

### Western blot analysis.

Overnight cultures were normalized by OD_600_. Samples were resuspended in the same volume of sample buffer either containing β-mercaptoethanol (β-ME) for reduced blots or lacking the reducing agent for oxidized blots. Samples were boiled for 10 min and subjected to electrophoresis on an SDS-PAGE gel (8% for LptD and 10% for all other proteins). Proteins were transferred to a nitrocellulose membrane (GE Healthcare, Amersham, United Kingdom). Immunoblotting was carried out using rabbit polyclonal antisera raised against LamB/OmpA/MBP (1:25,000), OmpA/OmpC/OmpF (1:10,000), LptD (1:10,000), BamA (1:25,000), or GroEL (1:10,000; Sigma). Donkey anti-rabbit IgG–horseradish peroxidase secondary antibody (GE Healthcare) was used at a dilution of 1:10,000 for all immunoblots.

### β-Galactosidase assay.

Measurement of β-galactosidase activity to quantitate σ^E^ activity was performed as previously described (60). The protocol was adapted to include the following changes: overnight cultures were grown in M63 minimal glucose medium at 30°C. Cultures were diluted 1:100 in fresh M63 minimal glucose and grown until late exponential phase (OD_600_, ∼0.8 to 1.0) at 30°C.

### Plasmid construction.

The following strategy was used to construct pGRG25Modular::*bamA*. pGRG25 was digested with NotI (61). The native promoter, defined as 1,000 bp upstream of *bamA*, and the *bamA* open reading frame were amplified from a wild-type MC4100 (Table S3) genomic preparation (Qiagen DNeasy Blood & Tissue kit). The kanamycin resistance cassette flanked by FLP recombination target (FRT) sites was amplified from a genomic preparation of a Keio allele (48, 49). The primers used to amplify this cassette included a spacer containing an ApaI restriction site to construct a modular system. The P*_bamA_*::*bamA* and FRTKanFRT fragments were incorporated into pGRG25/NotI using Gibson assembly (New England BioLabs). The construct was integrated into the chromosome using the protocol as previously described (61).

### Genetic linkage analysis.

Selective pressure was quantitated by cotransduction of linked *bamE*::*kan nadB*::Tn*10* markers into the indicated strains. P1*vir* carrying *bamE*::*kan nadB*::Tn*10* was transduced into BH17, BH26, BH359, and BH379, selecting for Tet^r^ transductants. Tet^r^ transductants were screened for Kan^r^ to identify transductants carrying both alleles. The cotransduction frequency was quantitated as the number of Tet^r^ Kan^r^ transductants over the total number of screened transductants (*n *=* *300). The total number of screened colonies represents three separate transductions.

### Availability of data.

The mass spectrometry proteomics data have been deposited to the ProteomeXchange Consortium and can be accessed with identifier PXD012335.
