# Supplementary material for: The Synthetic Phenotype of ΔbamB ΔbamE Double Mutants Results from a Lethal Jamming of the Bam Complex by the Lipoprotein RcsF
Source: mBio. 2019 May 21;10(3):e00662-19. doi: 10.1128/mBio.00662-19 (PMC6529638; doi:10.1128/mBio.00662-19)
Supplement: TABLE S1 [file mBio.00662-19-st001.docx]

**Table S1: GO Term analysis of significantly upregulated proteins identified by quantitative proteomics.***

| **Term** | **Number of Genes** | **Fold Enrichment** | **p-value** |
| --- | --- | --- | --- |
| Cellular response to osmotic stress | 4 | 11.4 | 3.4E-3 |
| Translational elongation | 3 | 10.2 | 3.0E-2 |
| Antibiotic transport | 3 | 10.2 | 3.0E-2 |
| Protein insertion into membrane | 4 | 9.8 | 5.7E-3 |
| Lipopolysaccharide transport | 4 | 8.5 | 8.8E-3 |
| Cellular response to antibiotic | 3 | 8.5 | 4.3E-2 |
| Cellular water homeostasis | 3 | 8.5 | 4.3E-2 |
| Colanic acid biosynthetic process | 6 | 7.9 | 5.6E-4 |
| Protein transport by the Sec complex | 3 | 7.3 | 5.8E-2 |
| Protein targeting | 3 | 7.3 | 5.8E-2 |
| Gram-negative-bacterium-type cell  outer membrane assembly | 5 | 7.1 | 3.8E-3 |
| Intracellular protein transmembrane  transport | 4 | 6.2 | 2.3E-2 |
| Peptidoglycan metabolic process | 4 | 6.2 | 2.3E-2 |
| Aerobic electron transport chain | 3 | 5.7 | 9.3E-2 |
| Cation transmembrane transport | 4 | 4.6 | 5.3E-2 |
| Chromosome segregation | 4 | 4.6 | 5.3E-2 |
| Potassium ion transport | 4 | 3.8 | 8.3E-2 |
| Drug transmembrane transport | 4 | 3.6 | 9.5E-2 |
| Protein autophosphorylation | 5 | 3.6 | 4.8E-2 |
| Peptidyl-histidine phosphorylation | 5 | 3.6 | 4.8E-2 |
| Regulation of cell shape | 10 | 3.5 | 1.7E-3 |
| Carbohydrate transmembrane transport | 5 | 3.4 | 5.4E-2 |
| Proteolysis | 7 | 3.3 | 1.6E-2 |
| Response to drug | 5 | 3.3 | 6.1E-2 |
| Peptidoglycan biosynthetic process | 8 | 3.0 | 1.4E-2 |
| Lipopolysaccharide biosynthetic process | 8 | 2.8 | 2.0E-2 |
| Cell wall organization | 10 | 2.3 | 2.4E-2 |
| Response to antibiotic | 10 | 1.9 | 7.2E-2 |

*GO term biological process enrichment analysis on significantly upregulated proteins (>0.95) identified in the Δ*bamB*Δ*bamE* double mutant as compared to wild-type. Terms are ranked in descending order of fold enrichment. Analysis was performed using DAVID Bioinformatics Software (1, 2).

**References**

1. **Huang DW**, **Sherman BT**, **Lempicki RA**. 2009. Systematic and integrative analysis of large gene lists using DAVID bioinformatics resources. Nat Protoc **4**:44–57.

2. **Huang DW**, **Sherman BT**, **Lempicki RA**. 2008. Bioinformatics enrichment tools: paths toward the comprehensive functional analysis of large gene lists. Nucleic Acids Research **37**:1–13.
