# Supplementary figures and images for: The Synthetic Phenotype of ΔbamB ΔbamE Double Mutants Results from a Lethal Jamming of the Bam Complex by the Lipoprotein RcsF
Source: mBio. 2019 May 21;10(3):e00662-19. doi: 10.1128/mBio.00662-19 (PMC6529638; doi:10.1128/mBio.00662-19)

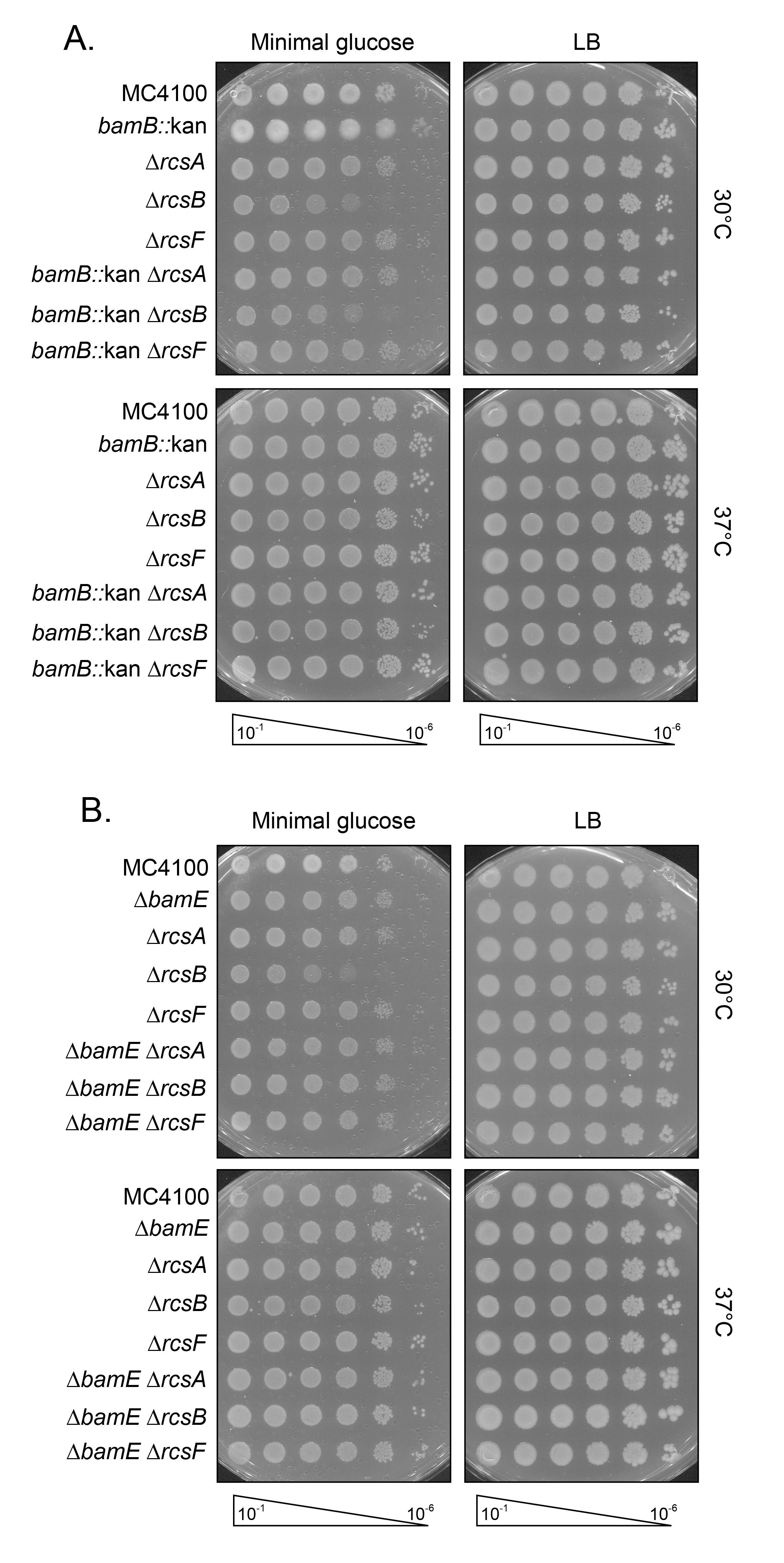

Supplement: FIG S1 [file mBio.00662-19-sf001.tif]

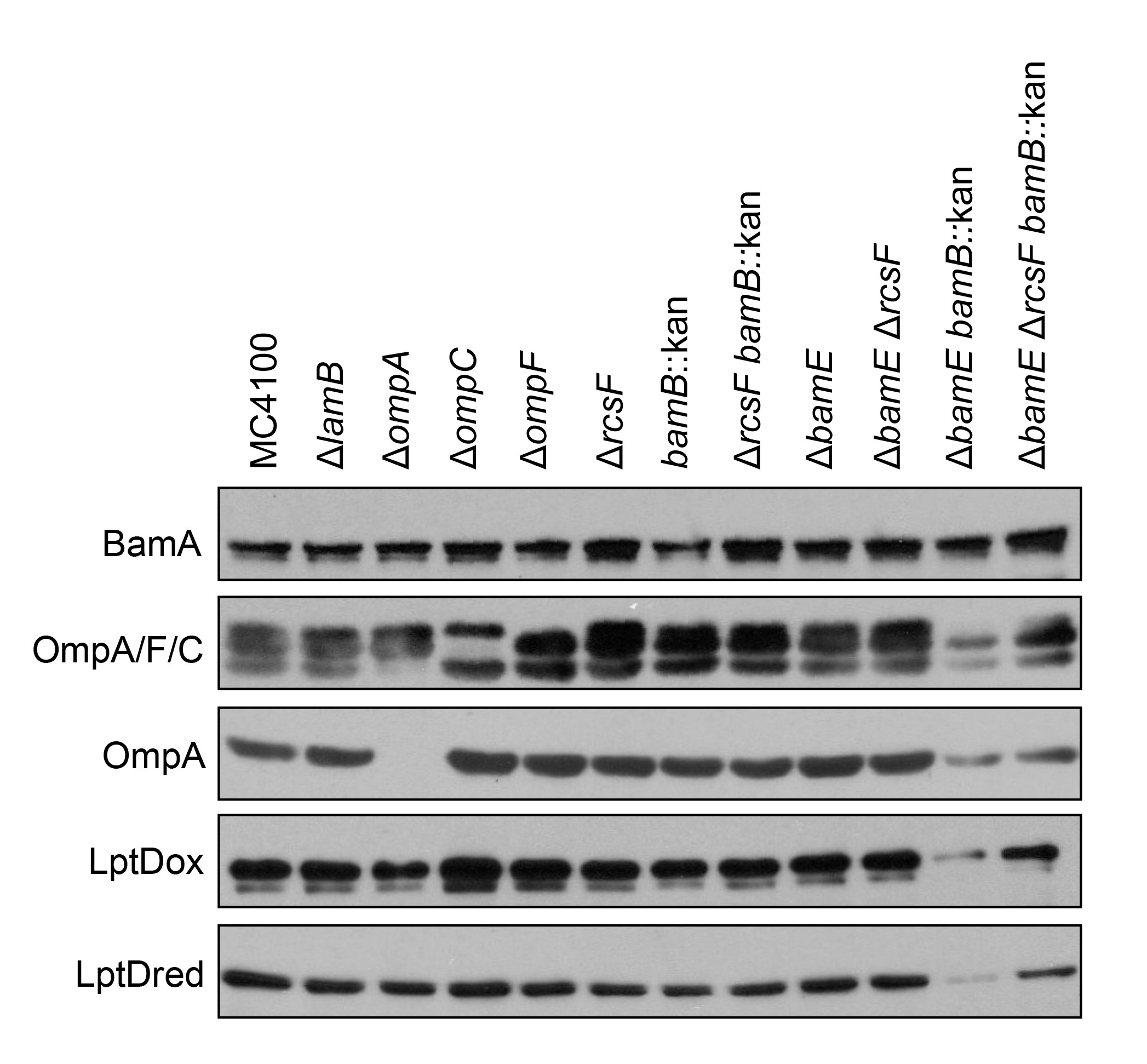

Supplement: FIG S2 [file mBio.00662-19-sf002.tif]

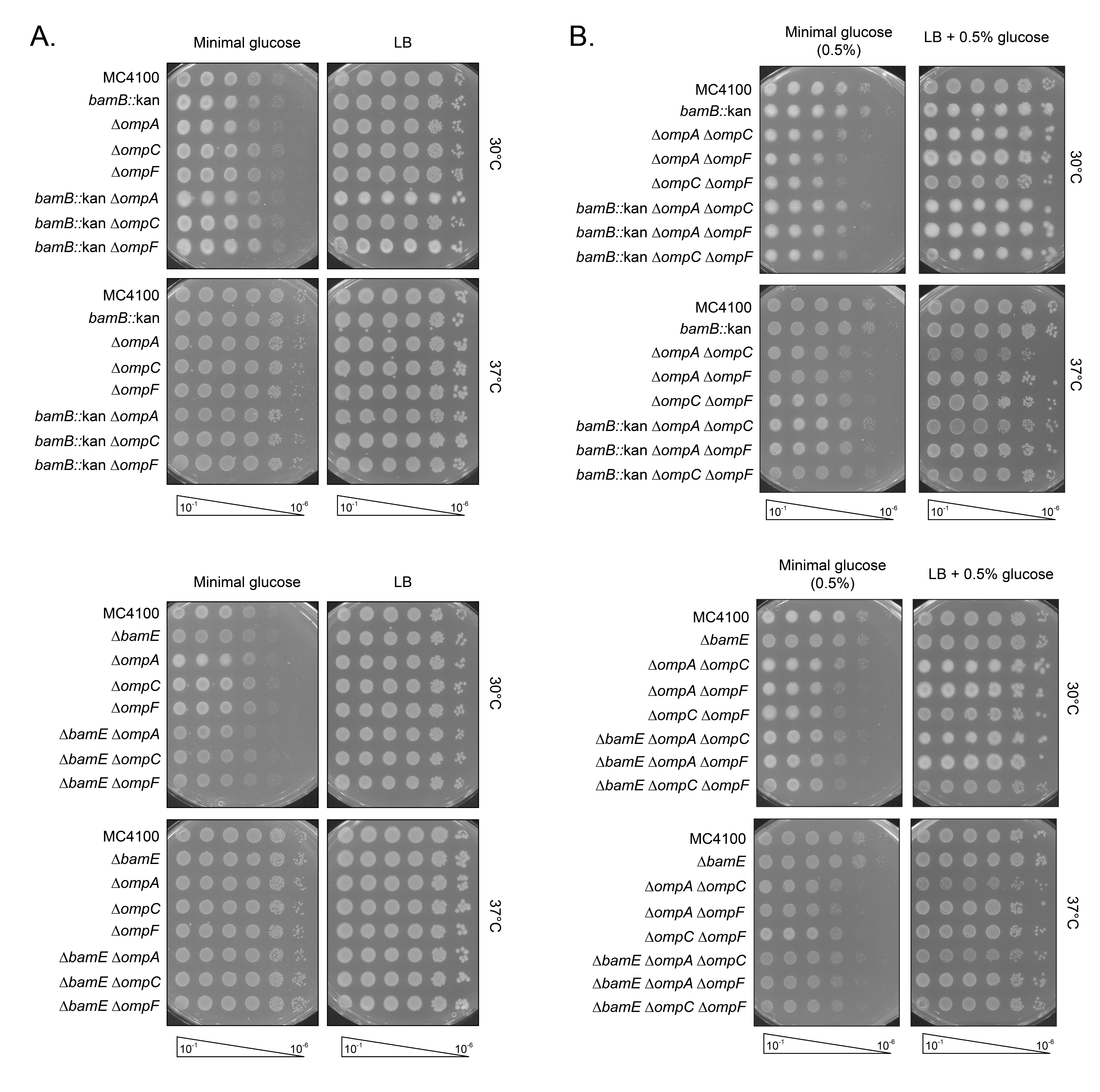

Supplement: FIG S3 [file mBio.00662-19-sf003.tif]

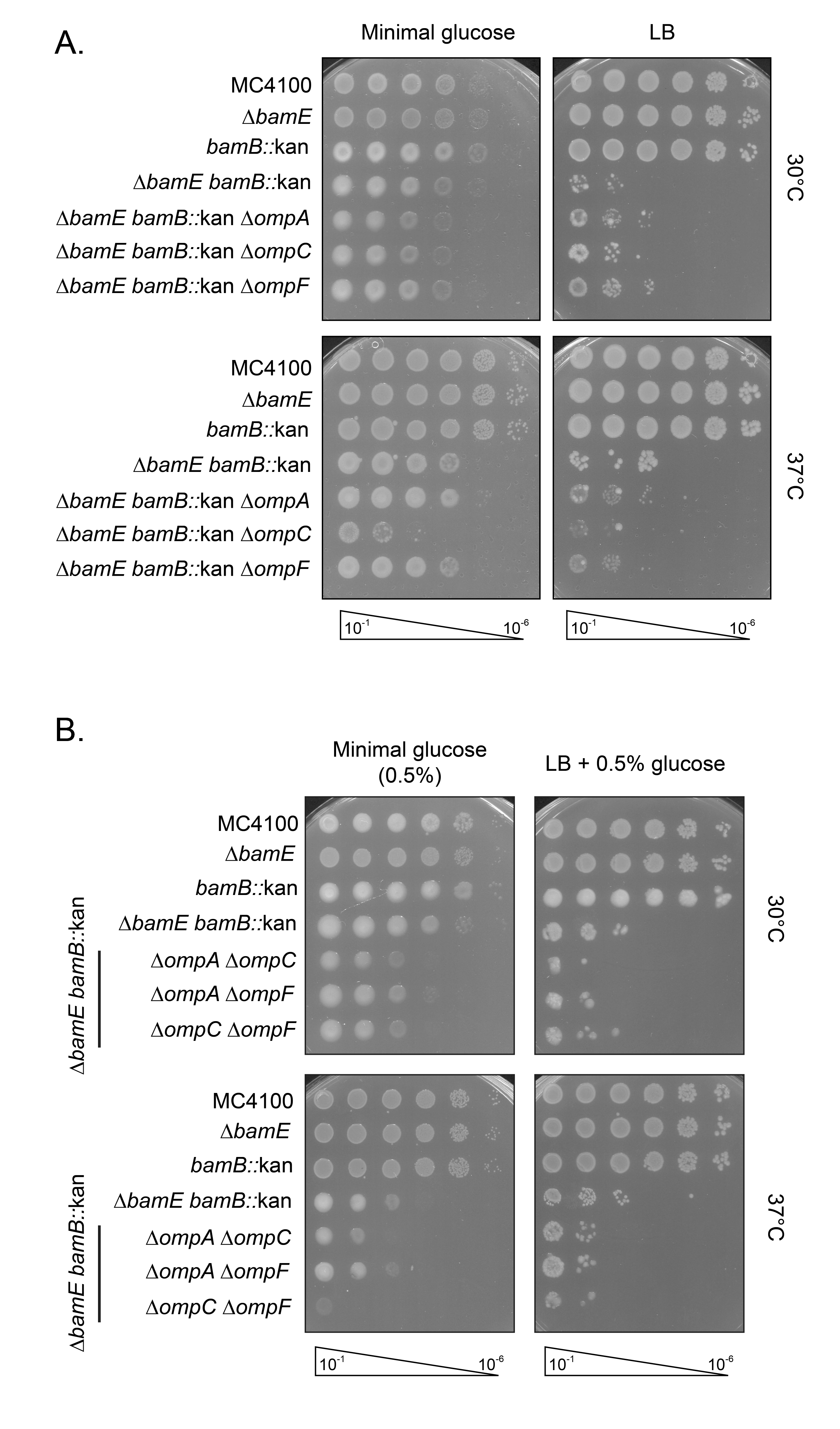

Supplement: FIG S4 [file mBio.00662-19-sf004.tif]

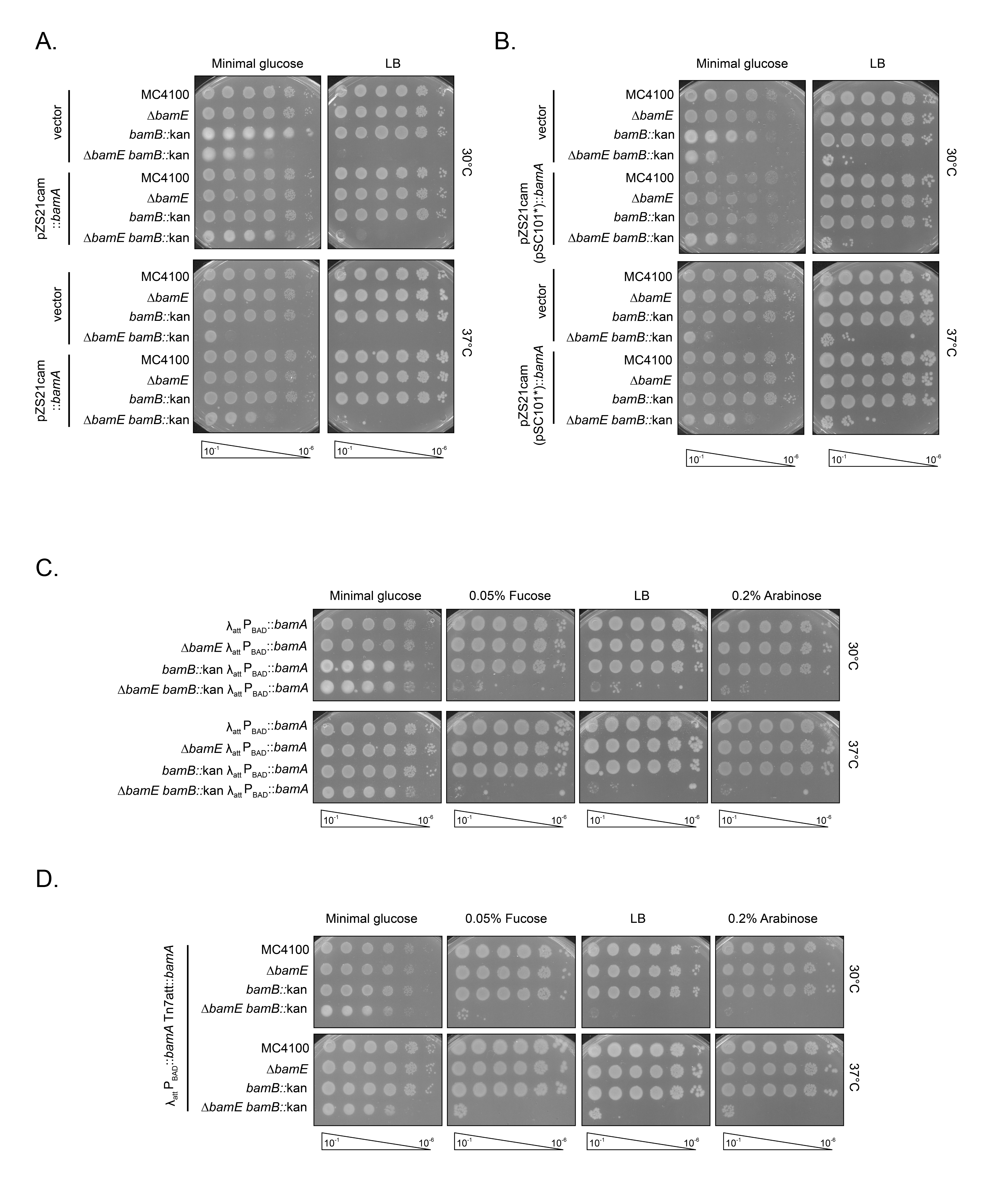

Supplement: FIG S5 [file mBio.00662-19-sf005.tif]
