# Supplementary material for: The Synthetic Phenotype of ΔbamB ΔbamE Double Mutants Results from a Lethal Jamming of the Bam Complex by the Lipoprotein RcsF
Source: mBio. 2019 May 21;10(3):e00662-19. doi: 10.1128/mBio.00662-19 (PMC6529638; doi:10.1128/mBio.00662-19)
Supplement: TABLE S3 [file mBio.00662-19-st003.docx]

**Table S3: Strains, plasmids, and oligonucleotides**

| *E. coli* K-12 strains | Genotype and relevant features | Reference |
| --- | --- | --- |
| MC4100 | F-*araD139* (*argF-lac*)*U169* *rpsL150 relA1 flb5301 deoC1 ptsF25 thi* | (1) |
| JCM158 | MC4100 *ara^r/-^* | (2) |
| NR669 | λ_att_ *rpoH-P3::lacZ* | (3) |
| JAS544 | Δ*bamE* | (4) |
| JCM318 | λ_att_ P_BAD_::*bamA* | (2) |
| BH92 | *bamB::*Tn5KAN-I-SceI (*bamB*::kan) | (5) |
| BH709 | Δ*bamE bamB::*kan (Δ*bamE*Δ*bamB*) | This study |
| BH717 | Δ*bamE bamA_F494L_ yaeH*::cam *bamB*::kan  (Δ*bamE*Δ*bamB bamA_F494L_*) | This study |
| BH735 | Δ*bamE* Δ*rcsF* | This study |
| BH828 | Δ*bamE* Δ*rcsF* *bamB*::kan (Δ*bamE*Δ*bamB*Δ*rcsF*) | This study |
| BH957 | Δ*bamE* Δ*rcsA* | This study |
| BH958 | Δ*bamE* Δ*rcsB* | This study |
| BH961 | Δ*rcsF* *bamB*::kan | This study |
| BH972 | Δ*bamE* Δ*rcsA* *bamB*::kan (Δ*bamE*Δ*bamB*Δ*rcsA*) | This study |
| BH976 | Δ*bamE* Δ*rcsB* *bamB*::kan (Δ*bamE*Δ*bamB*Δ*rcsB*) | This study |
| BH1016 | MC4100 λ_att_ *rpoH-P3-lacZ* | This study |
| BH1032 | Δ*bamE* λ_att_ P_BAD_::*bamA* | This study |
| BH1058 | Δ*bamE* λ_att_ *rpoH-P3-lacZ* | This study |
| BH1059 | Δ*bamE* Δ*rcsF* λ_att_ *rpoH-P3-lacZ* | This study |
| BH1061 | Δ*bamE* Δ*ompA* | This study |
| BH1062 | Δ*bamE* Δ*ompC* | This study |
| BH1063 | Δ*bamE* Δ*ompF* | This study |
| BH1085 | Δ*ompA* | This study |
| BH1086 | Δ*ompC* | This study |
| BH1087 | Δ*ompF* | This study |
| BH1088 | Δ*bamE* Δ*ompA bamB*::kan (Δ*bamE*Δ*bamB*Δ*ompA*) | This study |
| BH1092 | Δ*bamE* Δ*ompC bamB*::kan (Δ*bamE*Δ*bamB*Δ*ompC*) | This study |
| BH1096 | Δ*bamE* Δ*ompF bamB*::kan (Δ*bamE*Δ*bamB*Δ*ompF*) | This study |
| BH1105 | Δ*rcsA* | This study |
| BH1107 | Δ*bamE* pZS21cam (empty) | This study |
| BH1108 | Δ*bamE* pZS21cam::*bamA* | This study |
| BH1113 | MC4100 pZS21cam (empty) | This study |
| BH1115 | *bamB::*kan pZS21cam (empty) | This study |
| BH1116 | Δ*bamE bamB*::kan pZS21cam (empty) | This study |
| BH1117 | MC4100 pZS21cam::*bamA* | This study |
| BH1119 | *bamB*::kan pZS21cam::*bamA* | This study |
| BH1120 | Δ*bamE bamB*::kan pZS21cam::*bamA* | This study |
| BH1129 | Δ*rcsB* | This study |
| BH1179 | Δ*rcsA bamB*::kan | This study |
| BH1180 | Δ*rcsB bamB*::kan | This study |
| BH1135 | Δ*rcsF* λ_att_ *rpoH-P3-lacZ* | This study |
| BH1136 | *bamB::*kan λ_att_ *rpoH-P3-lacZ* | This study |
| BH1138 | Δ*rcsF bamB::*kan λ_att_ *rpoH-P3-lacZ* | This study |
| BH1139 | Δ*bamE bamB::*kan λ_att_ *rpoH-P3-lacZ* | This study |
| BH1141 | Δ*bamE* Δ*rcsF* *bamB::*kan λ_att_ *rpoH-P3-lacZ* | This study |
| BH1162 | Δ*rseA* λ_att_ *rpoH-P3-lacZ* | This study |
| BH1179 | Δ*rcsA bamB*::kan | This study |
| BH1180 | Δ*rcsB bamB*::kan | This study |
| BH1214 | Δ*bamE bamB*::kan pZS21(pSC101*)cam empty | This study |
| BH1215 | Δ*bamE bamB*::kan pZS21(pSC101*)cam::*bamA* | This study |
| BH1220 | MC4100 pZS21(pSC101*)cam empty | This study |
| BH1221 | MC4100 pZS21(pSC101*)cam::*bamA* | This study |
| BH1222 | Δ*bamE* pZS21(pSC101*)cam empty | This study |
| BH1223 | Δ*bamE* pZS21(pSC101*)cam::*bamA* | This study |
| BH1224 | *bamB*::kan pZS21(pSC101*)cam empty | This study |
| BH1225 | *bamB*::kan pZS21(pSC101*)cam::*bamA* | This study |
| BH1233 | Δ*bamE bamB*::kan λ_att_ P_BAD_::*bamA* | This study |
| BH1240 | *bamB*::kan λ_att_ P_BAD_::*bamA* | This study |
| BH1358 | Tn7_att_::*bamA*FRT λ_att_ P_BAD_::*bamA* | This study |
| BH1359 | Δ*bamE* Tn7_att_::*bamA*FRT λ_att_ P_BAD_::*bamA* | This study |
| BH1379 | Δ*bamE bamB*::kan Tn7_att_::*bamA*FRT λ_att_ P_BAD_::*bamA* | This study |
| BH1382 | *bamB*::kan Tn7_att_::*bamA*FRT λ_att_ P_BAD_::*bamA* | This study |
| BH1478 | Δ*bamE* Δ*rcsB* *bamB::*kan λ_att_ *rpoH-P3-lacZ* | This study |
| BH1479 | Δ*bamE bamA_F494L_ yaeH*::cam *bamB::*kan λ_att_ *rpoH-P3-lacZ* | This study |
| BH1508 | Δ*ompA* Δ*ompC* | This study |
| BH1509 | Δ*ompA* Δ*ompF* | This study |
| BH1510 | Δ*ompC* Δ*ompF* | This study |
| BH1511 | Δ*bamE* Δ*ompA* Δ*ompC* | This study |
| BH1512 | Δ*bamE* Δ*ompC* Δ*ompF* | This study |
| BH1522 | Δ*ompA* Δ*ompF bamB*::kan | This study |
| BH1523 | Δ*ompC* Δ*ompF bamB*::kan | This study |
| BH1524 | Δ*ompA* Δ*ompC bamB*::kan | This study |
| BH1525 | Δ*bamE* Δ*ompC* Δ*ompF bamB*::kan (Δ*bamE*Δ*bamB*Δ*ompC*Δ*ompF*) | This study |
| BH1526 | *bamE* Δ*ompA* Δ*ompC bamB*::kan (Δ*bamE*Δ*bamB*Δ*ompA*Δ*ompC*) | This study |
| BH1532 | Δ*bamE* Δ*ompA* Δ*ompF* | This study |
| **Plasmids** | **Description** | **Reference** |
| pET23/42 | pET23a(+) with multiple cloning site of pET42a(+), P_T7_-dependent expression vector | (6) |
| p*lptD* | pET23/42::*lptD* | (7) |
| p*lptD_Y721D_* | pET23/42::*lptD_Y721D_* | (8) |
| pZS21cam | Empty pZS21 vector with deleted ribosomal binding site with kanamycin cassette exchanged for chloramphenicol resistance cassette from pBAD33 vector | (9) (10) |
| pZS21cam::*bamA* | pZS21::*bamA* with chloramphenicol resistance | (11) |
| pZS21(pSC101*)cam | pSC101* origin from pZS*11 exchanged for pSC101 origin | (10) |
| pZS21(pSC101*)cam:::*bamA* | pSC101* origin from pZS*11 exchanged for pSC101 origin from pZS21cam::*bamA* | (10) |
| pGRG25Modular::*bamA* | Native promoter (1000bp upstream of *bamA*) driving *bamA* on pGRG25. Downstream of *bamA* ORF is a spacer containing the ApaI restriction site. Following the restriction site is a kanamycin resistance cassette flanked by FRT sites. | (12-14) |
| **Oligonucleotides** | **Sequence (5’ to 3’)** | **Information** |
| pZS21CamR Gibson F | CGTTCTGAACAAATCCAGATGGAGTTCTGAGGTC  AAATTTGCTTTCGAATTTCTGC | To amplify chloramphenicol resistance cassette from pBAD33 for Gibson assembly |
| pZS21CamR Gibson R | AGGTTAATGTCATGATAATAATGGTTTCTTAGGG  GGAATAAATACCTGTGACGGAAG | To amplify chloramphenicol resistance cassette from pBAD33 for Gibson assembly |
| pZS21 backbone F | CCCCTAAGAAACCATTATTATC | To amplify pZS21 backbone for Gibson assembly to change resistance cassette from kanamycin to chloramphenicol |
| pZS21 backbone R | TGACCTCAGAACTCCATCTG | To amplify pZS21 backbone for Gibson assembly to change resistance cassette from kanamycin to chloramphenicol |
| pZS21vector-Fwd | CTCGAGTCCCTATCAGTG | To amplify pZS21 vector for Gibson assembly to change pSC101 origin to pSC101* origin |
| pZS21vector-Rev | GTGAAGACGAAAGGGCCTC | To amplify pZS21 vector for Gibson assembly to change pSC101 origin to pSC101* origin |
| pSC101*-Fwd | CGAGGCCCTTTCGTCTTCACCCTAGGGTACGGGTTTTGC | To amplify pSC101* origin from pZS*11 |
| pSC101*-Rev | ATCACTGATAGGGACTCGAGGAGCTCGCTTGGACTCCTG | To amplify pSC101* origin from pZS*11 |
| Modular-PbamA-bamA-Fwd | TAATCAGATCCCTCAATAGCGTCCGGTGGTTGGCGAAATAG | To amplify native promoter and ORF of *bamA* from chromosome |
| Modular-PbamA-bamA-Rev | CTTCAAAAGGGCCCAAATTACCAGGTTTTACCGATGTTAAACTG | To amplify native promoter and ORF of *bamA* from chromosome |
| Modular-ApaI-KanFRT-Fwd | AACCTGGTAATTTGGGCCCTTTTGAAGTTCCTATACTTTCTAGAG | To amplify kanamycin cassette flanked by FRT sites from Keio allele |
| Modular-ApaI-KanFRT-Rev | CTCCTAGGTGCTCGAGTGGCTTTTGAAGTTCCTATTCTCTAG | To amplify kanamycin cassette flanked by FRT sites from Keio allele |

**References**

1. **Boyd D**, **Weiss DS**, **Chen JC**, **Beckwith J**. 2000. Towards Single-Copy Gene Expression Systems Making Gene Cloning Physiologically Relevant: Lambda InCh, a Simple *Escherichia coli* Plasmid-Chromosome Shuttle System. Journal of Bacteriology **182**:842–847.

2. **Malinverni JC**, **Werner J**, **Kim S**, **Sklar JG**, **Kahne D**, **Misra R**, **Silhavy TJ**. 2006. YfiO stabilizes the YaeT complex and is essential for outer membrane protein assembly in *Escherichia coli*. Mol Microbiol **61**:151–164.

3. **Button JE**, **Silhavy TJ**, **Ruiz N**. 2007. A suppressor of cell death caused by the loss of σ^E^ downregulates extracytoplasmic stress responses and outer membrane vesicle production in *Escherichia coli*. Journal of Bacteriology **189**:1523–1530.

4. **Rigel NW**, **Schwalm J**, **Ricci DP**, **Silhavy TJ**. 2012. BamE Modulates the *Escherichia coli* β-Barrel Assembly Machine Component BamA. Journal of Bacteriology **194**:1002–1008.

5. **Kang Y**, **Durfee T**, **Glasner JD**, **Qiu Y**, **Frisch D**, **Winterberg KM**, **Blattner FR**. 2004. Systematic Mutagenesis of the *Escherichia coli* Genome. Journal of Bacteriology **186**:4921–4930.

6. **Wu T**, **McCandlish AC**, **Gronenberg LS**, **Chng S-S**, **Silhavy TJ**, **Kahne D**. 2006. Identification of a protein complex that assembles lipopolysaccharide in the outer membrane of *Escherichia coli*. Proc Natl Acad Sci USA **103**:11754–11759.

7. **Chng SS**, **Ruiz N**, **Chimalakonda G**, **Silhavy TJ**, **Kahne D**. 2010. Characterization of the two-protein complex in *Escherichia coli* responsible for lipopolysaccharide assembly at the outer membrane. Proc Natl Acad Sci USA **107**:5363–5368.

8. **Lee J**, **Sutterlin HA**, **Wzorek JS**, **Mandler MD**, **Hagan CL**, **Grabowicz M**, **Tomasek D**, **May MD**, **Hart EM**, **Silhavy TJ**, **Kahne D**. 2018. Substrate binding to BamD triggers a conformational change in BamA to control membrane insertion. Proc Natl Acad Sci USA **115**:2359–2364.

9. **Guzman L-M**, **Belin D**, **Carson MJ**, **Beckwith J**. 1995. Tight Regulation, Modulation, and High-Level Expression by Vectors Containing the Arabinose PBAD Promoter. Journal of Bacteriology **177**:4121–4130.

10. **Lutz R**, **Bujard H**. 1997. Independent and tight regulation of transcriptional units in *Escherichia coli* via the LacR/O, the TetR/O and AraC/I1-I2 regulatory elements. Nucleic Acids Research **25**:1203–1210.

11. **Ricci DP**, **Hagan CL**, **Kahne D**, **Silhavy TJ**. 2012. Activation of the *Escherichia coli* β-barrel assembly machine (Bam) is required for essential components to interact properly with substrate. Proc Natl Acad Sci USA **109**:3487–3491.

12. **Baba T**, **Ara T**, **Hasegawa M**, **Takai Y**, **Okumura Y**, **Baba M**, **Datsenko KA**, **Tomita M**, **Wanner BL**, **Mori H**. 2006. Construction of *Escherichia coli* K-12 in-frame, single-gene knockout mutants: the Keio collection. Mol Syst Biol **2**:473–11.

13. **Datsenko KA**, **Wanner BL**. 2000. One-step inactivation of chromosomal genes in *Escherichia coli* K-12 using PCR products. Proc Natl Acad Sci USA **97**:6640–6645.

14. **McKenzie GJ**, **Craig NL**. 2006. BMC Microbiology. BMC Microbiol **6**:39–7.
