# Supplementary material for: The Synthetic Phenotype of ΔbamB ΔbamE Double Mutants Results from a Lethal Jamming of the Bam Complex by the Lipoprotein RcsF
Source: mBio. 2019 May 21;10(3):e00662-19. doi: 10.1128/mBio.00662-19 (PMC6529638; doi:10.1128/mBio.00662-19)
Supplement: TABLE S2 [file mBio.00662-19-st002.docx]

**Table S2: *lptD_Y721D_* Δ*bamE* synthetic lethality is suppressed by *bamA_F494L_****

| ***lptD* allele** | ***bamA* allele** | ***bamE::*kan *nadB::*Tn10**  **Co-transduction frequency (%)** |
| --- | --- | --- |
| *lptD^+^* | *bamA^+^* | 11 |
| *lptD_Y721D_* | *bamA* | 0 |
| *lptD^+^* | *bamA_F494L_* | 12 |
| *lptD_Y721D_* | *bamA_F494L_* | 7 |

*P1 vir lysates carrying *bamE*::kan *nadB*::Tn10 linked alleles were transduced into the indicated strains. Transductions were plated onto media containing tetracycline to select for successful transduction of *nadB*::Tn10 and ampicillin to maintain the plasmid encoding the *lptD* alleles. Tet^R^ transductants were then tested for Kan^R^ to calculate co-transduction frequency of both *bamE*::kan and *nadB*::Tn10 alleles. The co-transduction frequency represents three separate transductions.
